# When Segregation Hangs by a Thread

**DOI:** 10.1371/journal.pgen.1000371

**Published:** 2009-02-06

**Authors:** Giovanni Bosco

**Affiliations:** Department of Molecular and Cellular Biology, University of Arizona, Tucson, Arizona, United States of America; The University of North Carolina at Chapel Hill, United States of America

**Figure 1:**
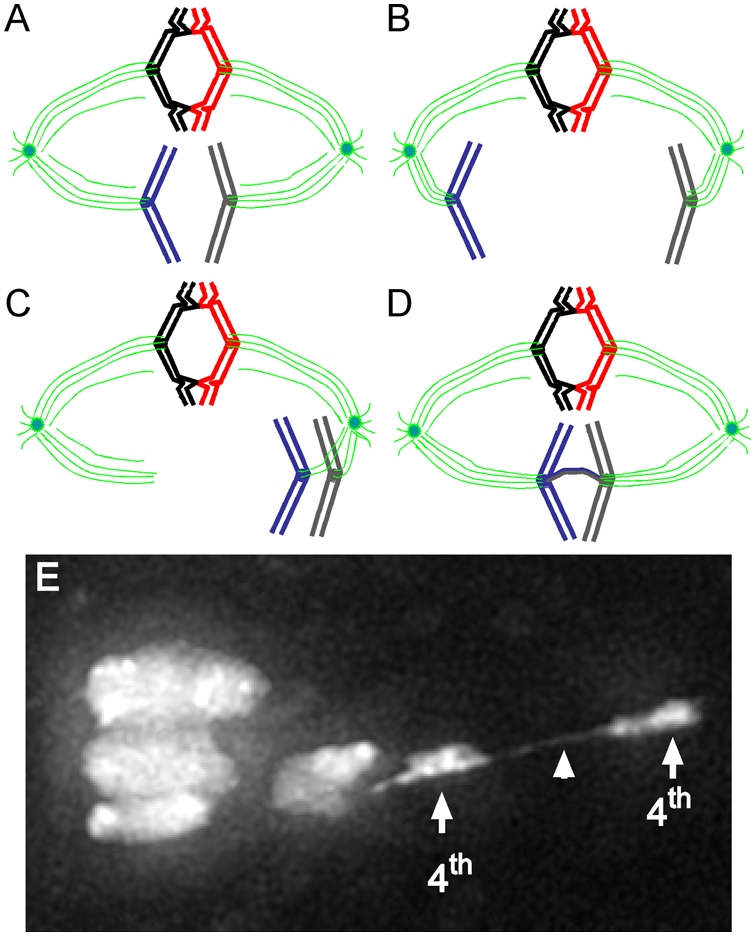
Model for achiasmate chromosome segregation. (A) Chiasmate homologs (red and black) are locked together by crossovers, whereas the sister chromatids are held together by cohesins (not shown). Achiasmate homologs (blue and gray) are not locked together by crossovers. Spindle (green) attachments to kinetochores (solid circles) are stabilized by tension created by pulling forces that draw chiasmate homologs to opposite poles. (B) Achiasmate chromosomes were thought not to be locked with their homologs and are able to move prematurely to one or the other spindle pole. (C) As shown by Hughes et al. [9], achiasmate homologs can be found on the same side of the metaphase plate. This is the first demonstration that this configuration can occur, and it suggests that achiasmate homologs can move in unison. (D) In addition, heterochromatic DNA threads between achiasmate homologs can be observed. These threads may provide chiasma-like function that lock homologs together and allow tension to be established between these nonexchange homologs. This tension is used by spindle forces to move achiasmate chromosomes along the spindle, orient them, make them join the mass of chiasmate chromosomes congressed at the metaphase plate, and ultimately ensures proper segregation. (E) An image of female *Drosophila* meiosis I chromosomes. In this oocyte from a female carrying a mutant allele of *ald* (the fly *mps1* homolog), a DAPI-staining thread (arrowhead) can be seen connecting the obligately achiasmate 4th chromosomes (arrows). Threads connecting achiasmate chromosomes were first observed in *ald* mutant females, but are also present (albeit less prominently) in wild-type oocytes. Based on chromosome orientation, the spindle is inferred to run from left to right, with the three chromosomes at the left defining the metaphase plate (image in (E) courtesy of W.D. Gilliland, S.E. Hughes, R.S. Hawley [9]).

The production of haploid gametes is essential for sexual reproduction in most eukaryotes. To achieve this haploid production, germline cells enter a specialized cell cycle called meiosis, in which two consecutive chromosome segregation events create gametes with half the number of chromosomes of diploid cells. Sister chromatids are held together by cohesins along the length of their arms and at their centromeres [1]. In meiosis, chromosomes seek yet another pairing partner—their homolog. DNA breaks, recombination, and ensuing crossovers between homologs result in a strong physical interaction that locks homologs together (Figure 1A). Cytologically, these crossovers can be observed as structures called chiasmata at discrete points connecting homologs [2]. Chromosomes that do not recombine are said to be achiasmate, because they do not have crossovers.

The combination of cohesins that bind sisters and chiasmata that lock homologs together results in a stable structure that opposes the poleward pull of microtubule spindles attached to centromeres (kinetochores) on both homologs [3]. When two chiasmate homologs are pulled to opposite poles, they engage in a dance, toward one pole or the other, until they come to rest at the middle of the two spindle poles, also known as the metaphase plate [4]. When all chromosomes congress to the metaphase plate and are stably attached to the spindle, the cell is in metaphase I; in oocytes this can be a prolonged arrest that awaits activation. The dissolution of sister-chromatid cohesion along the chromosome arms (but not at their centromeres) allows crossover chromosomes that were previously locked together in metaphase I to fly apart under the force of an anaphase I spindle. In meiosis II, centromeric cohesion holds sister chromatids together until the metaphase II–anaphase II transition, where sister chromatid separation and segregation creates haploid gametes [1].

Although crossovers are important for orienting homologs toward opposite poles of the spindle [3], they are not always essential for faithful segregation of homologs [5]. How these achiasmate homologs manage to segregate properly has been a long-standing puzzle [2]. Previous work in *Drosophila* by Hawley and colleagues provided substantial insights into this mysterious aspect of meiotic chromosome segregation. First, it was demonstrated that achiasmate segregation requires heterochromatic homology [6]. In a later study, using fluorescent in situ hybridization, it was shown that heterochromatic sequences themselves were involved in physical pairing of achiasmate homologous chromosomes [7]. These studies implicated heterochromatin in pairing of achiasmate homologs but did not address whether heterochromatin physically “locks” homologs together. It had been assumed that these achiasmate chromosomes could not significantly participate in the back-and-forth dance that brings all chiasmate chromosomes to the metaphase plate. This is because achiasmate chromosomes were believed to lack the locking connections that chiasmate chromosomes enjoy, and therefore it was assumed they could not provide any force to oppose their one functional spindle attachment. However, recent observations by Gilliland et al. demonstrated that achiasmate chromosomes do indeed join chiasmate chromosomes at the metaphase plate [8]. This raised the question as to how achiasmate chromosomes can be brought to join the main mass of the metaphase chromosomes if they cannot participate in spindle attachment-detachment cycles and re-orientation thought to be responsible for this congression.

In a study from the Hawley group in the January issue of *PLoS Genetics*, Hughes, Gilliland and colleagues show that achiasmate chromosomes also participate in the back-and-forth dance along the metaphase spindle that is characteristic of locked chromosomes being positioned on the metaphase plate [9]. By visualizing live *Drosophila* female meiotic chromosomes, the authors provide an unprecedented view of dramatic and dynamic achiasmate chromosome movement. Achiasmate homologs can be seen together on the same side of the metaphase plate, a configuration that had not been previously known to occur (Figure 1C and 1E). This is important because it suggests that achiasmate homologs can move together, as if they were locked. What is more astonishing is the clear demonstration that achiasmate chromosomes can actually be connected to one another by heterochromatic DNA threads. These DNA threads provide the first mechanistic insights into the dynamic movements and faithful segregation of achiasmate chromosomes. The authors suggest that these heterochromatic threads contribute substantially to a stable physical connection between achiasmate homologs. It is likely that these threads perform a chiasma-like function. Support for the idea that chromosomal threads could provide a sufficient chiasma-like locking force comes from studies in the crane fly, where severing a chromosome arm from its spindle-attached kinetochore causes this chromosomal fragment to traverse the metaphase plate and rejoin its thread-connected homolog [10]. Thus, movement of achiasmate chromosomes to one spindle pole can be opposed by a force exerted on its thread-locked achiasmate homolog attached to the opposite pole (Figure 1D).

These observations now raise more questions: First, what are these threads and how are they made? The authors suggest that stalled replication forks stimulate recombinational repair between satellite sequences of the homologs. Do heterochromatin protein–protein interactions contribute to the initiation and/or stability of these DNA threads? Second, can the occurrence of these threads be shown to change the efficiency of proper segregation of achiasmate chromosomes? If DNA threads provide chiasma-like functions, then either eliminating or stabilizing such threads should change the level of achiasmate non-disjunction. In order for thread-connected homologs to segregate properly in meiosis I, the threads must be removed. How is this done? Third, how common are these DNA threads, and do different systems use them as chiasma-like structures to hold homologs in a locked position? Male meiosis in *Drosophila* proceeds without chiasmata, and perhaps this system also uses DNA threads as a homolog-linking mechanism. Indeed, DNA threads have been observed linking the X and Y chromosomes [11]. DNA threads involving the mostly heterochromatic 4th chromosome have also been recently observed [12]. Lastly, what are the implications for the function of heterochromatin? If these heterochromatic threads are left unresolved, breakage might lead to gene conversion within peri-centric satellite sequences. DNA repair associated with the thread may also cause expansion and contraction of satellite repeats, which, over time, may lead to significant variation in heterochromatin content and homogenization of nonrepeat sequences within heterochromatin. Measurable differences in heterochromatin content have been documented for many *Drosophila* species [13]. Are these heterochromatic threads the mechanism driving satellite expansion and contraction, and do changes in satellite repeat content affect gene expression?

Answers to these and other questions await more detailed analysis of heterochromatic thread structure and regulation. Like all seminal studies revealing novel features of basic processes, the current study by Hawley and colleagues [9] raises more questions than it answers. What is certain is that staying connected to your homolog is important, and sometimes just a thread of a connection makes all the difference.
